# Population Pharmacokinetic and Pharmacogenetic Analysis of Nevirapine in Hypersensitive and Tolerant HIV-Infected Patients from Malawi

**DOI:** 10.1128/AAC.02069-13

**Published:** 2014-02

**Authors:** Laura Dickinson, Masautso Chaponda, Daniel F. Carr, Joep J. van Oosterhout, Johnstone Kumwenda, David G. Lalloo, Munir Pirmohamed, Robert S. Heyderman, Saye H. Khoo

**Affiliations:** a Department of Molecular and Clinical Pharmacology, University of Liverpool, Liverpool, United Kingdom; b Department of Medicine, College of Medicine, University of Malawi, Blantyre, Malawi; c Malawi-Liverpool-Wellcome Trust Clinical Research Programme, College of Medicine, University of Malawi, Blantyre, Malawi; d Dignitas International, Zomba, Malawi; e Liverpool School of Tropical Medicine, Liverpool, United Kingdom

## Abstract

We modeled nevirapine (NVP) pharmacokinetics in HIV-infected Malawian patients to assess the relationship between drug exposure and patient characteristics, genetic polymorphisms, and development of hypersensitivity reaction (HSR). One thousand one hundred seventeen patients were prospectively recruited and followed for 26 weeks with multiple or single serum samples obtained in a subset of patients for NVP quantification. Single nucleotide polymorphisms (SNPs) within *CYP2B6* and *CYP3A4* genes were typed. Nonlinear mixed effects modeling was utilized to assess the influence of patient characteristics and host genetics on NVP apparent oral clearance (CL/F) and to explore the relationship between NVP CL/F and HSR. Published haplotype distributions were used to simulate NVP concentrations in Caucasians versus Africans. One hundred eighty patients (101 female) were included in the model; 25 experienced HSR. No associations between patient demographics or HSR and NVP CL/F were evident. A significant relationship between *CYP2B6* c.983T>C and *CYP2B6* c.516G>T and NVP CL/F was observed (*P* < 0.01). NVP CL/F was reduced by 23% and 36% in patients with *CYP2B6* 983TT/516TT and 983TC/516GG or GT, respectively, compared to the reference genotype. Simulated exposures suggested similar proportions (13 to 17%) of patients with subtherapeutic NVP among Caucasians and an African population. Influence of *CYP2B6* polymorphisms on NVP CL/F in this population is in agreement with other reports. Our data indicate a lack of association between NVP exposure and HSR. Based on these data, dose optimization based solely on ethnicity (without individual gene testing) is unlikely to impact on risk of treatment failure or toxicity even in an African population with high carriage of poor metabolizer mutations.

## INTRODUCTION

Sub-Saharan Africa remains the region of the world most affected by HIV infection and is home to approximately two-thirds of all people living with HIV (1). In Malawi, antiretroviral therapy (ART) has been scaled up to reach nearly half a million individuals (http://www.hivunitmohmw.org/). Here, as in most other national ART programs in sub-Saharan Africa, a public health approach has been deployed to maximize health gains for the population. Most individuals (∼90%) receiving ART are on first-line, nevirapine (NVP)-containing regimens and are managed using a combination of clinical monitoring and symptom-driven laboratory observations.

Between 6 and 10% of patients receiving NVP develop cutaneous eruptions or liver injury, which can sometimes be severe and occasionally fatal (2, 3). The risk of such hypersensitivity reactions (HSRs) in African patients remains poorly characterized and may differ from those of cohorts in developed countries because national and WHO policies allow initiation of NVP-based therapy at higher CD4 counts. The relationship between drug exposure and development of NVP HSR is unclear, but previous studies have reported higher plasma drug exposures in black African and Thai patients (4) and higher rates of liver toxicity in patients receiving once-daily NVP than in those receiving twice-daily NVP, presumably due to higher maximum concentrations attained after dosing (5).

NVP is metabolized by cytochrome P450 enzymes CYP3A4 and CYP2B6 (6). Single nucleotide polymorphisms (SNPs) in the genes encoding both enzymes have been shown to impact NVP pharmacokinetics in various populations (7–9). In addition, CYP3A5 (which shares 90% substrate specificity with CYP3A4) is more commonly expressed in African populations and may influence NVP exposure. We have previously reported an association between *CYP2B6* polymorphisms and body weight with NVP plasma exposure in patients recruited in the United Kingdom and Germany (10). These pharmacogenetic influences carry potential implications for dose optimization, management of drug-drug interactions, “forgiveness” for missed doses, and choice of partner drugs for coformulation. Individualized genetic testing is rarely feasible in resource-limited settings. However, if pharmacogenetic information can be used to ensure that the dosing and design of regimens and treatment policy are optimized for populations rather than for individuals, considerable public health benefit may accrue.

In this study, we sought to characterize NVP pharmacokinetics in a Malawian population receiving ART. A modeling approach was utilized to assess associations between individual factors (including multiple genetic influences across different loci as well as nongenetic covariates) and NVP plasma exposure and also examined the relationship between exposure and the subsequent development of drug hypersensitivity. Finally, through the implementation of mathematical simulations we aimed to evaluate the hypothesis that pharmacogenetic information can be utilized to optimize NVP dosing for populations without the requirement for individual genetic testing.

## MATERIALS AND METHODS

### Patients.

Between March 2007 and September 2008, 1,117 HIV-infected antiretroviral treatment-naive patients starting on NVP-based therapy were prospectively recruited at the Queen Elizabeth Central Hospital, Blantyre, Malawi, and followed for 26 weeks. The study received full ethical approval from the Liverpool School of Tropical Medicine Research Ethics Committee (Liverpool, United Kingdom) and the College of Medicine Research and Ethics Committee, University of Malawi (Blantyre, Malawi). All patients gave their written informed consent. Patients received nevirapine at a dose of 200 mg twice daily (200 mg once daily within the first 2 weeks of therapy; lead-in dose) as part of a generic combination tablet formulation also containing lamivudine and stavudine. Inclusion criteria for the study were age greater than 16 years, ability to give informed consent, WHO staging visit performed in the antiretroviral (ARV) clinic, and ability to attend all study visits for 6 months. Patients were started on ART if they were WHO clinical stage III or IV and/or their CD4 cell count was <250 cells/mm^3^. Patients were excluded if they were jaundiced at baseline, taking concomitant tuberculosis treatment or hepatotoxic drugs, or currently pregnant or if they had received previous antiretroviral therapy. Patients who did not adhere to the study protocol, transferred to district centers outside the Blantyre catchment area, or had poor compliance with treatment (i.e., missed >10 tablets per month) were withdrawn from the study.

A diagnosis of NVP hypersensitivity was made if NVP treatment was discontinued and any of the following were observed: (i) rash (widespread maculopapular rash without systemic manifestations and getting worse on treatment continuation), (ii) hypersensitivity syndrome (widespread rash and systemic manifestations such as fever, cough, or abnormal liver function tests [LFT]), (iii) Stevens-Johnson syndrome (SJS), (iv) toxic epidermal necrolysis (TEN), or (v) drug-induced liver injury manifesting with jaundice. In all cases, the temporal relationship between drug initiation and the occurrence of the reaction, the effect of drug cessation, and the possibility of other possible causes was assessed. The AIDS Clinical Trials Group (ACTG) grading for LFT and rash severity (11) and the Naranjo method for estimating the causality of the adverse reaction (12) were used. The treating clinician first made the diagnosis of hypersensitivity in relation to NVP intake, and an independent assessment was then made by a dermatologist.

### Pharmacokinetic sampling and drug analysis.

A mixture of rich and sparse pharmacokinetic sampling was performed in a subset of patients. For NVP-tolerant patients included in the rich sampling subset (*n* = 40, 20 female), drug intake was directly observed and timed on the day of sampling and administered under fed conditions followed by blood sampling predose (0 h) and 1, 2, 5, and 9 h postdose. Patients willing to give more blood samples and remain at the clinic for a longer period of time were selected for the rich sampling subset. For those undergoing random sampling during clinic visits (*n* = 140), 1 sample per patient was taken, and if hypersensitivity developed later in the study, a second sampling event was undertaken. Venous blood samples (10 ml) were drawn, and serum was isolated within 2 h of collection and then stored (−70°C) until being shipped to Liverpool, United Kingdom, for drug analysis.

Serum NVP concentrations were quantified by a fully validated high-pressure liquid chromatography–tandem mass spectrometry (HPLC-MS/MS) method modified from that reported by Else et al. (13). The lower limit of quantification was 0.518 mg/liter, and the assay was linear up to 10 mg/liter with a precision and bias of <10%. The laboratory participated in an external quality assurance program (International Interlaboratory Quality Control Program for Therapeutic Drug Monitoring in HIV Infection, Nijmegen, The Netherlands) with an acceptable performance.

### Genotype analysis.

DNA was extracted from whole blood using a salt precipitation protocol (14). SNPs within the *CYP2B6* and *CYP3A5* genes were typed using the matrix-assisted laser desorption ionization–time of flight (MALDI-TOF)-based Sequenom iPLEX system (Sequenom Inc., San Diego, CA, USA) according to the manufacturer's protocol. Sequence-specific PCR and extended-reaction oligonucleotides were obtained from Metabion GmbH (Martinsried, Germany). Multiplex assays were designed using the software available from Sequenom. Additionally, the *CYP2B6* c.785A>G (rs2279343) polymorphism of CYP2B6 was genotyped using an Assays-by-Design TaqMan SNP genotyping assay (Applied Biosystems, Foster City, CA, USA).

PCR mixtures (10 μl) consisted of 1× TaqMan Universal PCR master mix (Applied Biosystems), 1× assay mix (unlabeled PCR primers and TaqMan minor groove binding [MGB]), probes (6-carboxyfluorescein [FAM] and VIC labeled), and 20 ng DNA. PCR was performed using an Applied Biosystems 7700 real-time PCR system (Applied Biosystems). The following cycling conditions were used: 95°C for 1 min followed by 40 cycles of 92°C for 15 s and 60°C for 1 min. Allelic discrimination analysis and genotype calls were made with the ABI 7700 sequence detection system (Applied Biosystems).

### Data analysis.

Nonlinear mixed effects modeling was applied (NONMEM version VI 2.0, level 1.1, double precision; Icon Development Solutions, Ellicott City, MD, USA) (15) using first-order conditional estimation with interaction (FOCE-I). Model fit was assessed by statistical and graphical methods. The minimal objective function value (OFV; equal to −2 log likelihood) was used as a goodness-of-fit diagnostic with a decrease of 3.84 points corresponding to a statistically significant difference between hierarchical models (*P* = 0.05, χ^2^ distribution, 1 degree of freedom). Graphical diagnostics were generated with Microsoft Office Excel 2007 for Windows (Microsoft Corporation, Redmond, WA, USA). Standard errors of the estimates were determined with the COVARIANCE option, and individual Bayesian parameter and concentration estimates were determined by the POSTHOC option.

### Pharmacokinetic and covariate model building.

One- and two-compartment models with first- or zero-order absorption without and with lag time were assessed to determine the best structural model to fit the data. To describe residual variability proportional, additive and combined proportional-additive error models were evaluated.

Covariates that could potentially influence NVP pharmacokinetics, including age, body weight, body mass index (BMI), sex, hypersensitivity, CD4 cell count, and viral hepatitis were investigated in the model. As well as linearly, BMI and CD4 cell count were stratified categorically by their cutoffs of BMI of < or >18.5 kg/m^2^ and CD4 of < or >250 cells/mm^3^. For continuous variables (e.g., body weight), plots of covariates versus individual predicted pharmacokinetic parameters were performed to determine possible relationships. Each covariate was introduced separately and retained only if inclusion in the model produced a statistically significant decrease in OFV of 3.84 (*P* ≤ 0.05) and was biologically plausible. Once all relevant covariates were included, a backwards elimination step was performed and covariates were retained if removal from the model produced a significant increase in OFV (>6.63 points; *P* ≤ 0.01, χ^2^ distribution, 1 degree of freedom).

### Pharmacogenetic model building.

SNPs considered for inclusion into the genetic model were CYP3A5*6 (SNP1), CYP3A5*3 (SNP2), *CYP2B6* c.983T>C (SNP3), *CYP2B6* c.516G>T (SNP4), and *CYP2B6* c.785A>G (SNP5). These specific allelic variants confer loss of function compared to the homozygous wild type.

Univariate analysis was performed for each SNP to test whether there was an association with NVP apparent oral clearance (CL/F); for inclusion into the model, the same statistical criteria were applied to the genetic data as were used for the demographic data outlined above.

### Model evaluation.

To assess the final model, a visual predictive check was performed. One thousand patients were simulated using the fixed and random effects defined by the final model with the SIMULATION SUBPROBLEMS option of NONMEM. From the simulated data, a 90% prediction interval (P5 to P95) was constructed, and observed data from the original data set were superimposed. At least 90% of data points falling within the prediction interval was indicative of an adequate model.

## RESULTS

### Patients.

In total, 180 HIV-infected patients were included in the pharmacokinetic model (101 female). This subset of patients was selected to represent sex, hypersensitivity, and viral hepatitis phenotypes of the larger data set (*n* = 1,117). As this was a substudy within the larger prospective study, no power calculations were performed. Median (range) age, weight, BMI, and CD4 cell count (all at start of ART) and duration of therapy at time of sampling were 34 years (21 to 62), 54 kg (35 to 94), 20 kg/m^2^ (15 to 38), 156 cells/mm^3^ (1 to 812), and 6 weeks (1 to 26), respectively. Twenty-five patients were hypersensitive (60% female; *n* = 18 rash, *n* = 3 jaundice, *n* = 4 SJS), 3 of whom developed a hypersensitivity reaction following the first pharmacokinetic sample. Overall, 23 patients were coinfected with hepatitis (hepatitis B virus [HBV], *n* = 12; HCV, *n* = 5; HBV/HCV, *n* = 6). All patients received the standard dose of NVP (200 mg twice daily), with the exception of 3 patients who were in the 2-week induction phase of 200 mg once daily.

### Pharmacokinetic sampling.

Of the 40 NVP-tolerant patients in whom rich sampling was performed, 35 also had 1 to 2 additional random samples drawn. In sparsely sampled patients, an additional sample was taken if hypersensitivity developed following the initial sampling day (*n* = 3). Time postdose ranged between 0.42 and 28 h; three samples taken 360 h (*n* = 1) and 312 h (*n* = 2) postdose were undetectable (below the lower limit of quantification; 0.518 mg/liter) and excluded from the analysis. A total of 383 samples were included with a median (range) NVP serum concentration of 5.7 mg/liter (1.2 to 25.5).

### Pharmacokinetic and covariate model.

A one-compartment model with first-order absorption best described the data; it was parameterized by NVP CL/F, apparent volume of distribution (*V/F*), and absorption rate constant (*k_a_*). Model fit was not significantly improved by the sequential addition of sample data from weeks 1 to 3 (to account for effects of autoinduction) but improved without fixing of *k_a_* compared with a fixed value of 1.66 h^−1^ as previously estimated (4) (ΔOFV, −12.0). In addition to interindividual variability (IIV) on CL/F, addition of IIV on *V/F* or *k_a_* or inclusion of an absorption lag time did not improve the model. Interoccasion variability (IOV) on CL/F significantly improved the model fit (ΔOFV, −82.5). IIV and IOV were described by exponential models, and residual error was described by a proportional model. Inclusion of a bioavailability factor (F1) to account for poor compliance in the sparse sampling data set did not improve the fit (ΔOFV, −0.5). The model failed to converge with inclusion of separate error models for sparse and rich data. NVP CL/F was described by the equation CL/F_*ij*_ = θ_1_ × exp(η_*i*_ + κ_*ij*_), where CL/F_*ij*_ illustrates NVP CL/F of the *i*th individual on the *j*th occasion, θ_1_ is the population parameter estimate, η_*i*_ is the IIV assumed to have a mean of zero and variance of ω^2^, and κ_*ij*_ is the IOV assumed to have a mean of zero and variance of π^2^.

Residual error was described as *Y* = *F* × (1 + ε_1_), where *Y* is the observed NVP concentration, *F* is the predicted concentration, and ε_1_ is the proportional random effects, assumed to have a mean of zero and variance of σ_1_^2^.

Pharmacokinetic parameters of the basic model are summarized in Table 1. None of the patient characteristics evaluated, including experience of HSR, showed a significant association with NVP CL/F (ΔOFV of between −0.02 and −3.1).

**TABLE 1:** Nevirapine parameter estimates and standard errors obtained from the basic population pharmacokinetic model and the final pharmacokinetic/pharmacogenetic model (*n* = 180)^*a*^

Parameter (unit)	Basic model		Final model	
	Estimate	RSE^*b*^ (%)	Estimate	RSE^*b*^ (%)
CL/F (liters/h)	2.74	3.76	3.02	4.44
*V/F* (liters)	113	23.2	114	22.8
*k_a_* (h^−1^)	0.569	26.7	0.578	26.1
IIV CL/F (%)	33.5	26.9	33.2	27.4
IOV CL/F (%)	34.9	23.3	31.8	24.2
Covariates				
θ_1_ *CYP2B6* 983TT/516TT			−0.696	36.6
θ_2_ *CYP2B6* 983TC/516GG or GT			−1.08	22.3
Residual error				
Proportional (%)	13.1	17.0	13.1	17.0

a Abbreviations: CL/F, apparent oral clearance of nevirapine (basic model) or apparent oral clearance of nevirapine in patients with reference genotype wt_983T>C_/wt or het_516G>T_ (final model); *V/F*, apparent volume of distribution; *k_a_*, absorption rate constant; IIV, interindividual variability; IOV, interoccasion variability; RSE, relative standard error; SE, standard error; θ_1_, absolute change in CL/F for *CYP2B6* 983TT/516TT genotype compared to reference genotype (*CYP2B6* 983TT/516GG or GT); θ_2_, absolute change in CL/F for het_983T>C_/wt or het_516G>T_ genotype compared to reference genotype.

b RSE = (SE_estimate_/estimate) × 100.

### Pharmacogenetic analysis.

Pharmacogenetic data were missing for 23, 25, 23, 26, and 33 patients for SNPs 1 to 5, respectively, due to poor quality of the DNA or genotyping failure. In total, data for all 5 SNPs were not available for 21 patients, and for another 21 patients, data were missing for either 1 SNP or a combination of 2 SNPs; however, all patients were included. Genotypes were in Hardy-Weinberg equilibrium.

Allelic frequencies for heterozygote and homozygote mutants for CYP3A5*6 and CYP3A5*3 were 31.8 and 1.3% and 29.0 and 5.2%, respectively. No homozygote mutants (CC) were observed for *CYP2B6* c.983T>C, but the frequency of heterozygotes was 17.2% (TC). Heterozygote and homozygote mutant allelic frequencies for *CYP2B6* c.516G>T were 48.7% (GT) and 16.2% (TT), respectively, and those for *CYP2B6* c.785A>G were 44.2% (AG) and 15.0% (GG), respectively.

Each genotype was evaluated individually, and a separate random effect was included for each genotype and to accommodate the missing data as shown in the following: CL = CL_0_ + θ_1_GEN_1_(1 − MISS) + θ_2_GEN_2_(1 − MISS) + θ_3_MISS, where CL_0_ is the typical value of NVP CL/F for homozygote wild-type individuals; θ_1_, θ_2_, and θ_3_ are the absolute changes in NVP CL/F for heterozygotes and homozygote mutants (heterozygotes only for *CYP2B6* c.983T>C) and missing data, respectively, compared to homozygote wild-type patients; GEN_*i*_ is an indicator variable which takes the value of 1 for the *i*th genotype (i.e., GEN_1_, heterozygotes; GEN_2_, homozygote mutants) and otherwise takes the value 0; and MISS is an indicator variable for missing data, also taking the value of 1 or 0.

Following univariate analysis, *CYP2B6* c.983T>C, *CYP2B6* c.516G>T, and *CYP2B6* c.785A>G were significantly associated with NVP CL/F (ΔOFV, −16.9, −5.6, and −6.4, respectively). NVP CL/F was reduced by 0.958 liter/h (33.0%) in *CYP2B6* 983 heterozygotes (TC). The contribution of *CYP2B6* 785AG (heterozygote) appeared to be small (increase in CL/F of 0.00207 liter/h, 0.07%), and a decrease in CL/F of 0.641 liter/h (22.9%) was observed for homozygous mutants (GG). A reduced model combining wild type (785AA) and heterozygotes (785AG) produced a similar result (ΔOFV, −6.4; decrease in CL/F of 22.9% for homozygous mutants). NVP CL/F was decreased by 7.6% and 19.5% in *CYP2B6* 516 heterozygotes (GT) and homozygote mutants (TT), respectively.

### Multiple SNP analysis.

Based on the univariate analysis, pairwise combinations were evaluated in the model for SNPs with the most impact on NVP CL/F. Addition of *CYP2B6* c.785A>G genotypes in the reduced form (i.e., AA and AG combined) into a model containing *CYP2B6* c.983T>C significantly improved the fit (ΔOFV, −6.1). NVP CL/F was reduced by 29.1% and 34.5% in patients with *CYP2B6* 983TT/785GG and *CYP2B6* 983TC/785AA or AG, respectively. However, following a backwards elimination step the *CYP2B6* c.785A>G genotype was no longer significant.

**FIG 1:**
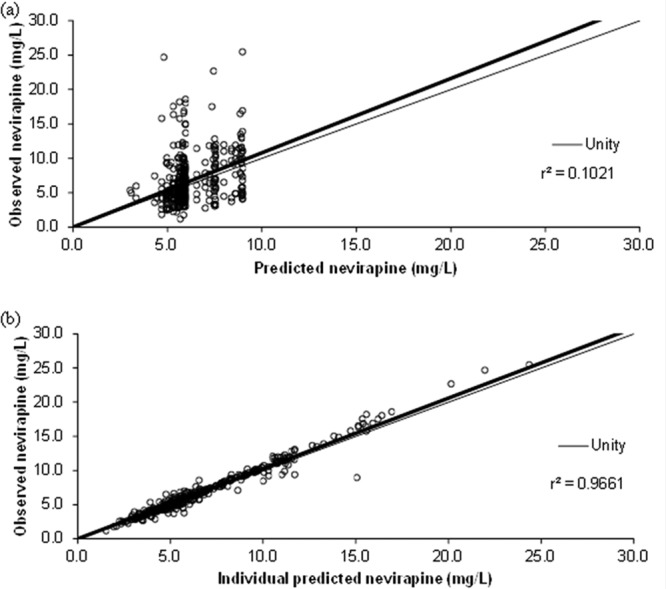
Goodness-of-fit plots for the final nevirapine pharmacokinetic-pharmacogenetic model (*n* = 180 patients) illustrating population predictions of nevirapine versus observed concentrations (a) and individual predictions of nevirapine versus observed concentrations (b). The fine line describes the line of unity, and the bold line describes the line of regression.

Addition of the *CYP2B6* c.516G>T SNP to the model containing the *CYP2B6* c.983T>C SNP significantly reduced NVP CL/F by 5.3%, 25.5%, 34.4%, and 39.5%, respectively, for the combinations *CYP2B6* 983TT/516GT, *CYP2B6* 983TT/516TT, *CYP2B6* 983TC/516GG, and *CYP2B6* 983TC/516GT, respectively (ΔOFV, −7.6). The change in NVP CL/F for the patients with *CYP2B6* 983TT/516GT was small (5.3%) compared to the reference genotype (*CYP2B6* 983TT/516GG), and so the *CYP2B6* 516GT genotypes were combined with the reference data (reduced model). This was still significant (ΔOFV, −7.0). NVP CL/F was decreased by 23.0% and 35.8% for patients with *CYP2B6* 983TT/516TT and *CYP2B6* 983TC/516GG or GT genotypes, respectively. Following backwards elimination, *CYP2B6* c.516G>T was retained in the model. Diagnostic plots of the final model, represented by population predicted nevirapine concentrations versus observed concentrations and individual model predicted concentrations versus observed nevirapine concentrations, are shown in Fig. 1, and model parameters are summarized in Table 1.

Of 383 measured concentrations, 24 (6.3%; measured between 0 and 9 h) were below the recommended minimum effective concentration (MEC) for NVP of 3.0 mg/liter (16–18). The concentrations corresponded to *CYP2B6* 983TT/516GG or GT (50.0%), *CYP2B6* 983TC/516TT (4.2%), and missing (45.8%) genotypes. Trough concentrations (*C*_trough_) (concentrations 12 h postdose) were not available for any of the patients included in the model and were therefore predicted for each patient at each occasion (*n* = 219). Median (range) individual predicted trough concentrations were 5.25 mg/liter (0.55 to 23.39), and 22/219 were <3.0 mg/liter. The majority of the concentrations below the MEC corresponded to *CYP2B6* 983TT/516GG or GT, and none were *CYP2B6* 983TC/516GG or GT genotypes.

### Model evaluation.

**FIG 2:**
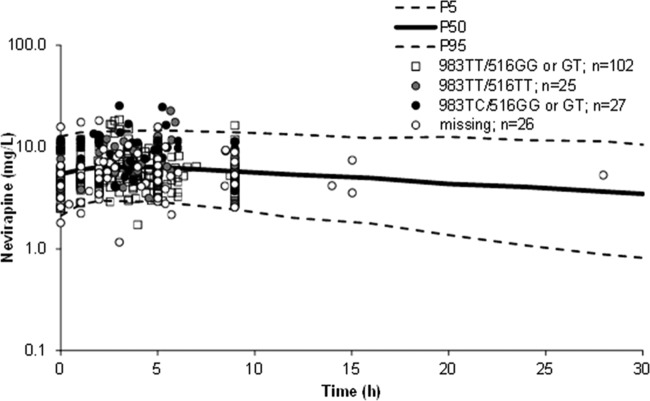
Nevirapine 90% prediction interval (P5 to P95) for the final model combining *CYP2B6* 983T>C and *CYP2B6* 516G>T genotypes from 1,000 simulated patients. The simulations were performed with a distribution of genotypes identical to that of the original data set. Observed data were superimposed (*n* = 383 concentrations; *n* = 180 patients).

Ninety percent prediction intervals (P5 to P95) were constructed from 1,000 simulated patients using the fixed and random effects of the final model containing both SNPs (*CYP2B6* 983T>C and 516G>T), and observed data were superimposed. Concentrations were simulated out until 30 h postdose so as to encompass all observed data (latest sample = 28 h postdose). The data were simulated so that the distribution of alleles was identical to that of the original data set. Of 383 observed concentrations, 4% were above P95 and 2% were below P5 (Fig. 2), indicative of an adequate model.

**FIG 3:**
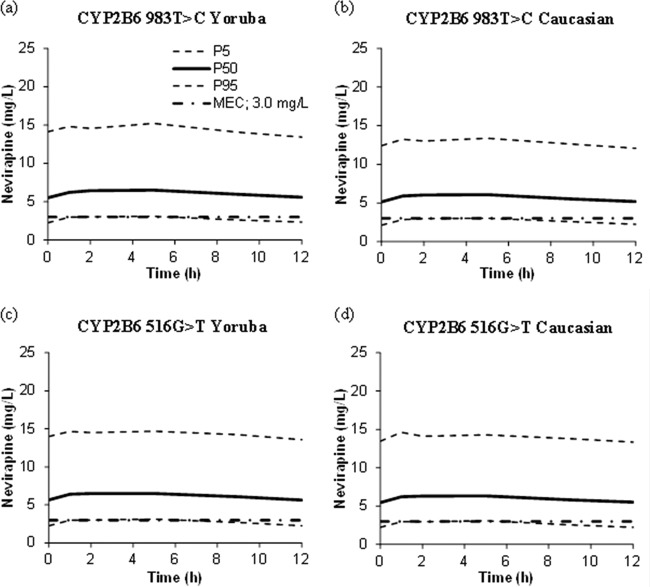
Nevirapine 90% prediction intervals generated from 1,000 simulated patients using the allelic frequencies of *CYP2B6* 983T>C and *CYP2B6* 516G>T in the Caucasian population and the Yoruba population (to represent Malawian individuals). (a) *CYP2B6* 983T>C, Yoruba; (b) *CYP2B6* 983T>C, Caucasian; (c) *CYP2B6* 516G>T, Yoruba; (d) *CYP2B6* 516G>T, Caucasian. The recommended minimum effective concentration (MEC) of nevirapine (3.0 mg/liter) is also shown (16, 22, 24).

In addition, models containing *CYP2B6* c.983T>C and *CYP2B6* c.516G>T separately as covariates were used to simulate NVP concentrations in a Caucasian population and a Yoruba population (*n* = 1,000 per group of individuals) based on the allele distributions provided by HapMap (http://www.hapmap.org). The Yoruba population from Nigeria was used as the closest available representation genetically to the Malawian population (Fig. 3) and has also been used as a comparator group for haplotype distributions for another cohort of Malawian patients (19). Based on the simulations, the 90% prediction intervals were similar between populations despite the decrease in NVP CL/F for *CYP2B6* 983TC (33%) and *CYP2B6* 516TT (20%) compared to wild type and even though the frequency of mutant alleles was higher in the African population (frequency of *CYP2B6* 983TT in the Yoruba population was included as missing data as patients in the original data set did not possess this genotype and, therefore, change in CL/F compared to wild type could not be determined). The Yoruba and Caucasian populations had similar percentages of simulated concentrations below the NVP MEC for both SNPs (*CYP2B6* c.983T>C, 17% versus 13%; *CYP2B6* c.516G>T, 15% versus 13%).

## DISCUSSION

We have developed and validated a pharmacokinetic model describing twice-daily NVP in a population of HIV-infected Malawian adults, which evaluates both demographic covariates and multiple pharmacogenetic influences. Consistent with previous reports, *CYP2B6* 516G>T and 983T>C polymorphisms were shown to be associated with NVP exposure (7–10).

These data suggest a lack of any direct relationship between drug exposure (as measured by CL/F) and development of NVP HSR. This is in keeping with the broadly accepted hypothesis that NVP HSR is idiosyncratic, rather than a concentration-dependent toxicity. Median individual predictions of CL/F, maximum concentrations, and *C*_trough_ for those without and with HSR were similar (2.9 versus 2.2 liters/h, 6.1 versus 5.8 mg/liter, and 5.2 versus 4.9 mg/liter, respectively). However, the number of patients with HSR was small compared to the number of NVP-tolerant individuals. Studies evaluating a relationship between plasma exposure of NVP and HSR are rare, and no previous study has prospectively attempted to look for a concentration-toxicity relationship in HSR. Formation of reactive intermediate metabolites, such as the hydroxyl metabolite 12-OH NVP (a potential cause of HSR), is likely to be influenced by a number of different factors such as host genetic susceptibility and environmental factors, in addition to drug exposure. Gender and CD4 count are recognized risks for NVP HSR (20), and we have also observed significant associations with human leukocyte antigen (HLA)-C*04:01 genotype and predisposition to NVP cutaneous reactions, namely, SJS/TEN, in this cohort (*n* = 117 hypersensitive versus *n* = 154 tolerant patients) (21). Moreover, a recent study by Sharma and coworkers provided evidence that 12-OH NVP sulfate formed in the skin was responsible for NVP-induced rash (22).

CYP3A4 and CYP3A5 share 90% sequence homology, and there is a significant overlap in substrate specificity (23). CYP3A5 polymorphisms may be of importance when considering African populations and metabolism of NVP, because CYP3A5 is more commonly expressed in Africans, particularly in comparison to Caucasians. CYP3A5*3 encodes a truncated, nonfunctional protein (24). The CYP3A5*3 allele is at a low frequency in African populations; however, a recent small study observed an association between CYP3A5*3 and NVP exposure (area under the concentration-time curve from 0 to 12 h [AUC_0-12_]) in 24 HIV-infected adults and children from Malawi (19). The association was evident following correction for *CYP2B6* 516G>T polymorphism and age, but surprisingly, CYP3A5*3 correlated with lower AUC_0-12_ rather than the expected higher exposure (19). CYP3A5*3 or indeed CYP3A5*6 did not show a significant relationship with NVP CL/F in the current cohort of HIV-infected Malawian patients, but allelic frequencies were similar to those reported by HapMap for the Yoruba population (present study versus HapMap: AA, 0.66 versus 0.68; AG, 0.29 versus 0.31; GG, 0.05 versus 0.01) and those observed in the previously published Malawi study (present study versus previous publication: AA, 0.66 versus 0.50; AG, 0.29 versus 0.50; GG, 0.05 versus 0.0). None of the patient characteristics investigated, such as weight, sex, and age, had a significant influence on NVP pharmacokinetic parameters. The relationship between NVP CL/F and *CYP2B6* 516G>T, although significant, described only 3% of the variability in NVP CL/F, which is consistent with a population analysis in HIV-infected patients from Cambodia (25). Following a further analysis of the Cambodian data, additional genetic variants described 11% of the variability in NVP CL/F (*CYP2B6* 516G>T, *CYP2B6* rs7251950, and *CYP2B6* rs2279343) (26). Furthermore, Liptrott and coworkers reported an independent association between a polymorphism in the efflux transporter ABCC10 (MRP7; rs2125739), along with *CYP2B6* 516G>T, BMI, and time postdose, and NVP plasma concentrations; however, the clinical implications of this relationship are currently unknown (27). Variability in NVP CL/F from the present study was unremarkable (∼30% for interindividual and interoccasion variability); however, inclusion of other factors that were not recorded, for example, other SNPs, may better predict NVP pharmacokinetics.

The homozygous mutant for *CYP2B6* 983T>C (983CC) was not present in this group of Malawian patients; however, the presence of the heterozygote (983TC) in combination with *CY2B6* 516GG or 516GT resulted in decreased NVP CL/F. Reduced NVP CL/F under these circumstances is potentially due more to the influence of *CYP2B6* 983T>C than to the *CYP2B6* 516G>T polymorphism, because individuals who are *CYP2B6* 983TC can exhibit either partial or full loss of function (internal data). Of 219 model predicted *C*_trough_, 10% were below the recommended MEC of 3.0 mg/liter for NVP (16–18). All of these patients possessed wild-type genotypes for both *CYP2B6* 983T>C and 516G>T or wild-type 983T>C with heterozygous 516G>T. All patients with 983TC experienced *C*_trough_ above the MEC, suggesting that with just one mutant allele for this polymorphism, patients may be more likely to suppress virus, although this remains speculative without supportive pharmacodynamic data. Choices of pharmacokinetic parameters to be used for the MEC threshold and whether the same cutoffs should be used globally are often debated. NVP MEC is based on studies performed mainly in Caucasian patients, and given the differences in NVP concentrations often observed between different populations, it is questionable whether the same MEC should be applied to African populations; however, an alternative is not yet available. Viral load measurements were not taken as part of this study, and changes in CD4 count from baseline to study completion (6 months on therapy) were not monitored. However, of the full cohort of 1,117 patients recruited and started on NVP-based therapy, 103 patients died (9%), 3 of which were a result of HSR, and 9% did not complete the 6-month follow-up due to withdrawal of consent, protocol violations, transfer to another clinic outside the Blantyre catchment area, or becoming lost to follow-up.

Despite the reduction in NVP CL/F with *CYP2B6* 516G>T and 983T>C SNPs in this cohort, based on simulations with Yoruba and Caucasian genotype frequencies, the influence at a population level was minimal. Moreover, similar proportions of *C*_trough_ were below the recommended NVP MEC for both populations (13 to 17%), although carriage of poor metabolizer mutations was higher in the simulated African population. Potentially, this could be a result of the particular genotypes studied here having little impact on NVP variability, or other factors, or indeed SNPs, could play a more important role in describing exposure for this compound. Potentially, further genetic studies are warranted to help identify other SNPs that may be of importance regarding antiretroviral pharmacokinetics in African populations. However, individualized genetic testing or therapeutic drug monitoring is unlikely to be widely deployed to guide personalized prescribing in resource-limited settings.

Our findings indicate that without the availability of individual gene testing and therapeutic drug monitoring, NVP dosing does not be need to be adapted on the basis of ethnicity, as this would not reduce the risk of treatment failure (based on the recommended NVP MEC) or prevent toxicity.
